# What Exempts from Conscription

**Published:** 1863-08

**Authors:** 


### WHAT EXEMPTS FROM CONSCRIPTION.

1. Manifest imbecility or insanity.

2. Epilepsy. For this disability, the statement of the drafted man is insufficient, and the fact must be established by the duly affidavit of a physician of good standing, who has attended him in a convulsion.

3. Paralysis, general, or of one limb, or chorea—their existence to be adequately determined.

4. Acute or organic disease of the brain or spinal cord; of the heart or lungs; of the stomach or intestines; of the liver or spleen; of the kidneys or bladder—sufficient to have impaired the general health, or so well marked as to leave no reasonable doubt of the man's incapacity for military service.

5. Confirmed consumption; cancer; aneurism of the large arteries.

6. Inveterate and extensive disease of the skin, which will necessarily impair his efficiency as a soldier.

7. Decided feebleness of the constitution, whether naturally or acquired.

8. Scrofula, or constitutional syphilis, which has resisted treatment, and seriously impaired his general health.

9. Habitual and confirmed intemperance, or solitary vice, in a degree sufficient to have materially enfeebled the constitution.

10. Chronic rheumatism, unless manifested by positive change of structure, wasting of the affected limb, or puffiness or distortion of the joints, does not exempt. Impaired motion of the joints, and contraction of the limbs, alleged to arise from rheumatism, and in which the nutrition of the limb is not manifestly impaired, are to be proven by examination, while in a state of anæsthesia, induced by ether only.

11. Pain, whether simulating headache, neuralgia in any of its forms, rheumatism, lumbago, or affections of the muscles, bones, or joints, is a symptom of disease so easily pretended, that it is not to be admitted as a cause for exemption, unless accompanied with manifest derangement of the general health, wasting of a limb, or other positive sign of disqualifying local disease.

12. Great injuries, or diseases of the skull, occasioning impairment of the intellectual faculties, epilepsy, or other manifest nervous or spasmodic symptoms.

13. Total loss of sight; loss of sight of right eye; cataract; loss of crystalline lens of right eye.

14. Other serious diseases of the eye, affecting its integrity and use, e. g., chronic ophthalmia, fistula lachrymalis, ptosis (if real), ectropion, entropion, &c. Myopia, unless very decided, or depending upon some structural changes in the eye, is not cause for exemption.

15. Loss of nose; deformity of nose so great as seriously to obstruct respiration. Ozœna, dependent upon caries in progress.

16. Complete deafness. This disability must not be admitted on the mere statement of the drafted man, but must be proved by the existence of positive disease, or by other satisfactory evidence. Purulent otorrhœa.

17. Caries of the superior and inferior maxillary, of the nasal or palate bones, if in progress; cleft palate (bony); extensive loss of substance of the cheeks, or salivary fistula

18. Dumbness; permanent loss of voice, not to be admitted without clear and satisfactory proof.

19. Total loss of tongue; mutilation or partial loss of tongue, provided the mutilation be extensive enough to interfere with the necessary use of the organ.

20. Hypertrophy or atrophy of the tongue, sufficient in degree to impair speech or deglutition. Obstinate chronic ulceration of the tongue.

21. Stammering, if excessive and confirmed; to be established by satisfactory evidence, under oath.

22. Loss of a sufficient number of teeth, to prevent proper mastication of food, and tearing the cartridge.

23. Incurable deformities, or loss of part of either jaw, hindering biting of the cartridge, or proper mastication, or greatly injuring speech; anchylosis of lower jaw.

24. Tumors of the neck, impeding respiration or deglutition; fistula of the larynx or trachea; torticollis, if of long standing and well marked.

25. Deformity of the chest, sufficient to impede respiration, or to prevent the carrying of arms and military equipments. Caries of the ribs.

26. Deficient amplitude, and power of expansion of chest. A man 5 feet 3 inches (minimum standard height for the Regular Army) should measure not less than thirty inches in circumference, immediately above the nipples, and have an expansive mobility of not less than two inches.

27. Abdomen grossly protuberant; excessive obesity. Hernia, either inguinal or femoral, ventral, umbilical, &c.

28. Artificial anus; stricture of the rectum; prolapsus ani; fistula in ano is not a positive disqualification, but may be so if extensive, or complicated with visceral disease.

29. Old and ulcerated internal hæmorrhoids, if in degree to impair the man's efficiency. External hæmorrhoids are no cause for exemption.

30. Total loss, or nearly total loss of penis; epispadius or hypospadius, at the middle or near the root of the penis.

31. Incurable, permanent, organic stricture of the urethra, in which the urine is passed drop by drop, or complicated by disease of the bladder. Recent or spasmodic stricture of the urethra does not exempt. Urinary fistula.

32. Incontinence of urine being a disease frequently feigned, and of rare occurrence, is not, of itself, a cause for exemption. Stone in the bladder, ascertained by the introduction of the metallic catheter, is a positive disqualification.

33. Loss, or complete atrophy of both testicles, from any cause; permanent retention of one or both testicles within the inguinal canal, but voluntary retraction does not exempt.

34. Confirmed or malignant sarcocele; hydrocele, if complicated with organic diseases of the testicle. Varicocele and circocele are not, in themselves, disqualifying.

35. Excessive anterior and posterior curvature of the spine; caries of the spine.

36. Loss of an arm, fore-arm, hand, thigh, leg, or foot.

37. Wounds, fractures, tumors, atrophy of the limb, or chronic diseases of the joints or bones, that would impede marching, or prevent continuous muscular exertion.

38. Anchylosis, or irreducible dislocation of the shoulder-, elbow-, wrist-, hip-, knee- or ankle-joint.

39. Muscular or cutaneous contractions, from wounds or burns, in degree sufficient to prevent useful motion of a limb.

40. Total loss of a thumb, loss of ungual phalanx of right thumb.

41. Total loss of any two fingers of same hand.

42. Total loss of index finger of right hand.

43. Loss of the first and second phalanges of the fingers of the right hand.

44. Permanent extension and permanent contraction of any finger except the little finger; all the fingers adherent or united.

45. Total loss of either great toe; loss of any three toes on the same foot; all the toes joined together.

46. The great toe crossing the other toes, with great prominence of the articulation of the metatarsal bone, and first phalanx of the great toe.

47. Overriding or superposition of all the toes.

48. Permanent retraction of the last phalanx of one of the toes, so that the free border of the nail bears upon the ground; or flexion at a right angle of the first phalanx of a toe upon a second, with anchylosis of this articulation.

49. Club-feet, splay feet, where the arch is so far effaced that the tuberosity of the scaphoid bone touches the ground, and the line of the station runs along the whole internal border of the foot, with great prominence of the inner ankle; but ordinary large, ill-shaped, or flat feet, do not exempt.

50. Varicose veins of inferior extremities, if large and numerous, having clusters of knots, and accompanied with chronic swellings or ulcerations.

51. Chronic ulcers; extensive, deep and adherent cicatrices of lower extremities.

52. *Stature.* If the height of the drafted man be greater than six feet four inches, or less than five feet three inches, he is disqualified for military service. The height in all doubtful cases to be determined by accurate measurement, the recruit being made to stand erect on his bare feet, and care being taken that he neither increases nor lessens his stature by voluntary effort.

53. No certificate of a physician or surgeon is to be received in support of the claims of drafted men for exemption from military service, unless the facts and statements therein set forth are affirmed or sworn to before a civil magistrate competent to administer oaths.

